# Pneumococcal Immunization Strategies for High-Risk Pediatric Populations Worldwide: One Size Does Not Fit All

**DOI:** 10.3390/vaccines9121390

**Published:** 2021-11-24

**Authors:** Theano Lagousi, Ioanna Papadatou, Petros Strempas, Elena Chatzikalil, Vana Spoulou

**Affiliations:** 1 Immunobiology Research Laboratory and Infectious Diseases Department “MAKKA”, First Department of Paediatrics, “Aghia Sophia” Children’s Hospital, Athens Medical School, 11527 Athens, Greece; joanna-pap@hotmail.com (I.P.); vspoulou@med.uoa.gr (V.S.); 2 Athens Medical School, University Research Institute of Maternal and Child Health and Precision Medicine, National and Kapodistrian University of Athens, 11527 Athens, Greece; 3 First Department of Paediatrics, “Aghia Sophia” Children’s Hospital, Athens Medical School, 11527 Athens, Greece; petrosstrempas@gmail.com (P.S.); ehatzikali@gmail.com (E.C.)

**Keywords:** *Streptococcus pneumoniae*, high-risk children, pneumococcal vaccines, high-income countries, middle- and low-income countries

## Abstract

Despite the significant reduction in pneumococcal disease due to pneumococcal vaccines, protection of vulnerable high-risk individuals, especially pediatric populations, remains a great challenge. In an effort to maximize the protection of high-risk children against pneumococcal disease, a combined schedule that includes both conjugate and polysaccharide vaccines is recommended by several countries in the developed world. On the other hand, middle- and low-income countries do not have in place established policies for pneumococcal immunization of children at risk. Pneumococcal conjugate vaccines, despite their benefits, have several limitations, mainly associated with serotype replacement and the wide range of serotype coverage worldwide. In addition, PPV23-impaired immunogenicity and the hyporesponsiveness effect among populations at risk have been well-documented. Therefore, the added value of continuing to include PPV23 in vaccination schedules for high-risk individuals in the years to come remains to be determined by monitoring whether the replacing/remaining serotypes causing IPD are covered by PPV23 to determine whether its benefits outweigh its limitations. In this review, we aim to describe serotype distribution and vaccine efficacy data on pneumococcal disease in the pre- and post-PCV implementation era among high-risk children in both developed and developing countries, assessing the optimization of current recommendations for their vaccination against pneumococcal disease.

## 1. Introduction

*Streptococcus pneumoniae* (*S. pneumoniae*) is a leading cause of mucosal diseases (e.g., otitis media, sinusitis, and non-bacteremic pneumonia) as well as invasive infections (e.g., meningitis and bacteremia) with considerable morbidity and mortality worldwide, comprising a major public health problem [1]. Young children under 2 years of age and the elderly are more vulnerable to pneumococcal infection [2,3]. In addition, individuals with weakened immune systems are at higher risk for pneumococcal infection, associated hospitalizations, and related mortality. Only minor differences exist regarding the high risks for pneumococcal disease when considering different settings worldwide. According to the CDC and the American Academy of Pediatrics, high-risk conditions for pneumococcal disease include cerebrospinal fluid leak, cochlear implants, diabetes, HIV infection or immunodeficiencies (congenital, acquired, or secondary to medications), anatomic or functional asplenia, sickle cell disease and other hemoglobinopathies, neoplasms, and chronic diseases including chronic heart, lung, kidney, or liver diseases, with incidence rates more than 50 times higher than those among children of the same age without these conditions [3,4,5,6]. Similarly, the latter conditions as well as previous repetitive episodes of invasive pneumococcal disease (IPD), trisomy 21, and solid organ transplants are considered high risk factors for pneumococcal disease by the Department of Health of the Australian Government [4,5]. In England, chronic liver disease, immunosuppression, and chronic respiratory diseases, including asthma, are the most common IPD risk factors among children 2–15 years of age [6,7]. Asthma is not currently considered a high-risk condition for pneumococcal infection in the United States, unless treated with high-dose oral corticosteroid therapy [8,9].

The number of patients at high risk requiring protection against IPD is persistently increasing owing to increasing numbers of people with underlying medical conditions and the elderly population worldwide. Pneumococcal infection is often more severe in such high-risk individuals than in immunocompetent subjects [10,11,12]. Currently, recorded resistance to common antimicrobials further impedes the successful treatment of pneumococcal infections [13,14]; therefore, optimal protection of high-risk groups against pneumococcal disease, especially children, through vaccination remains challenging.

Currently, two types of pneumococcal vaccines, both targeting capsular polysaccharides, are licensed: the 23-valent pneumococcal polysaccharide-based vaccine (PPV23) and the 10- and 13-valent pneumococcal conjugate vaccines (PCV-10 and PCV-13, respectively) (Table 1). PPV23 primarily induces a T-cell-independent immune response, is poorly immunogenic in children under 2 years of age, and elicits neither an immune memory response nor herd protection since it has no effect on pneumococcal carriage [15,16,17]. Conjugation of pneumococcal polysaccharides to a highly immunogenic carrier protein turned polysaccharide-based vaccines from T-cell-independent to T-cell-dependent antigens, enhancing their immunogenicity and extending the duration of the vaccine-induced immune response. In addition, conjugation of capsular polysaccharides with a carrier protein induces both arms of the immune response (B- and T-cell response), leading not only to a systemic immune response but also to the production of IgA and IgG antibodies in saliva, enhancing the mucosal immune response and mucosal immune memory as well [17]. PPV23 is effective among children older than 2 years of age and young adults, while it is less effective among elderly adults [18]. Age-related ineffectiveness may be due to age-associated changes in the antibody repertoire and/or a reduction in IgM antibody production due to changes in B cell subpopulations as well as to impaired T-cell immunity [18]. On the other hand, PCVs are immunogenic in both children younger than 2 years of age and the elderly.

Following routine PCV administration in North America and Europe, a rapid and dramatic decline in vaccine-type (VT) invasive pneumococcal disease (IPD) and nasopharyngeal carriage was recorded not only among vaccinated children (direct effects) but also among unvaccinated children and adults through reduced transmission of vaccine serotypes (indirect effects) [19,20]. Reductions were significantly enhanced following the replacement of PCV7 by the higher-valency conjugate pneumococcal vaccines PCV10 and PCV13. PCVs’ impact in low-income countries is less obvious as many countries in Africa and East Asia did not introduce PCV10 or PCV13 into their Expanded Program of Immunization (EPI) before 2011, financially supported by the Global Alliance for Vaccines and Immunization (GAVI), the vaccine alliance. South Africa, Rwanda, and Gambia were the exceptions, implementing PCV7 in 2009 and PCV13 afterwards. Thus, the initial data come from the latter countries, showing a significant decline in VT-IPD, pneumococcal pneumonia, and hospitalizations for severe pneumonia among vaccinated individuals [21].

For more than two decades, PPV23 has been recommended for the protection of immunocompromised individuals and the elderly against pneumococcal disease [22]. Following PCVs’ licensure, a combined schedule that includes PCV13 followed by PPV23 has been recommended in many countries in Europe and North America in an effort to enhance the protection of high-risk individuals against *S. pneumoniae*. Combined PCV13/PPV23 immunization schedules are expected to combine the establishment of an immunological memory with maximum serotype coverage. Current guidelines from the U.S. Centers for Disease Control and Prevention (CDC) [8] and the American Academy of Pediatrics recommend four doses of PCV13 (in a 3 + 1 schedule) and a dose of PPV23 at 2 years of age for children with conditions considered high risk for IPD or as soon as possible after a diagnosis of chronic illness is made after the age of 2 years. PPV23 vaccination is recommended for patients with asthma only if they are treated with high-dose oral corticosteroid therapy [8,9]. Among European countries, where the main vaccination schedules are 3 + 1 or 2 + 1, only Cyprus, France, Greece, Spain, and the United Kingdom recommend PPV23 vaccination additionally to the basic PCV13 scheme for the high-risk children [23]. Regarding low-income countries, the vast majority of these regions in Africa and South-East Asia do not have in place established policies for pneumococcal immunization of high-risk children, although IPD cases and associated deaths are higher compared with Europe and North America, mainly due to the higher prevalence of comorbidities that increase an individual’s vulnerability to pneumococcal disease in these settings [24,25,26].

The net benefits of pneumococcal vaccination are at least partially offset by several limitations. Both PPV23 and PCVs are serotype-based vaccines and therefore they elicit only serotype-specific protection. Emergence of replacement serotypes has repeatedly occurred after their introduction, even after the use of expanded-valency PCVs, albeit to a lesser extent [9,10,11,12]. Serotypes 8, 12F, and 33F are the most common serotypes responsible for the replacement phenomenon [27]. Increases in serotype 12F have been recorded in certain European countries (England, Wales, France, and Germany), while serotype 33F has been one of the most important causes of NT-IPD among children in several countries (the United States, Israel, and France) [27,28]. Vaccine failures reported following PCV13 implementation remain an important challenge mainly associated with serotypes 3 and 19A [29,30]. In addition, PCVs are less efficient against non-IPD (including otitis media, sinusitis, and non-bacteremic pneumonia) compared with IPD (e.g., meningitis, bacteremia, and bacteremic pneumonia) [20,31,32,33,34]. Moreover, their serotype coverage in developed countries is substantially higher compared with the developing world, where pneumococcal disease is caused by a wider spectrum of serotypes [35]. Remarkably, controversy regarding PPV23’s effectiveness among high-risk individuals raises several issues about the optimal use of polysaccharide vaccines in the context of combined vaccination schedules [36]. Most importantly, evidence of PPV23-induced immunological hyporesponsiveness has been well documented among high-risk individuals, representing a quite important issue in the developing world with a high prevalence of comorbidities [36]. Hyporesponsiveness is a phenomenon where vaccine recipients are unable to elicit an immune response that is higher or at least of the same magnitude as the primary response following repeated vaccinations [37]. The extent of the hyporesponsiveness impact upon vaccine protection remains unclear, but it is expected to be of greater importance in individuals with distinct immunocompromising conditions, where antibody levels are known to be already limited compared with the healthy [36]. Hyporesponsiveness was first described in the 1990s following vaccination with meningococcal polysaccharide vaccines and has been attributed to the immune tolerance induced by the vaccine polysaccharide antigens [38,39]. Since then, several studies have demonstrated the same phenomenon following pneumococcal polysaccharide vaccination [36].

Decisions to fund and implement vaccination recommendations in both the developed and the developing world require assessing data on epidemiology in conjunction with vaccine efficacy data in each region. The added value of continuing to include PPV23 in vaccination schedules for high-risk individuals in the years to come remains to be determined by monitoring whether the replacing/remaining serotypes causing IPD are covered by PPV23 to determine whether its benefits outweigh its limitations. In this review, we aim to describe serotype distribution and vaccine efficacy data on pneumococcal disease in the pre- and post-PCV implementation era among the high-risk children in both developed and developing countries, assessing the optimization of current recommendations for the vaccination of the high-risk children against pneumococcal disease. To this aim, an extensive PUBMED literature search was conducted using the terms “pneumococcal vaccines”, “high-risk children”, “immunocompromised children”, “vaccine efficacy”, “vaccine effectiveness”, “polysaccharide conjugate vaccines”, “PCV7”, “PCV10”, “PCV13”, “23-valent vaccine”, “23-valent pneumococcal capsular polysaccharide vaccine”, “Pneumovax 23”, “PPSV23”, “PPV23”, “Pneumo23”, and “pneumococcal polysaccharide vaccine”.

### 1.1. Current Burden of Disease and Serotype Epidemiology in High-Income Countries

Universal childhood pneumococcal vaccination with PCVs+/-PPV23 has significantly reduced the pneumococcal disease burden in high-income countries among individuals at risk, although residual pneumococcal disease remains significantly high among such vulnerable populations due to limited vaccine efficacy or high rates of replacement disease [27,40,41,42,43,44,45,46,47,48,49,50]. Data from several countries highlight the significant proportion of PPV23-only serotypes among the serotypes that emerged following PCV implementation (Table 2). In a recent study from the United States, during the post-PCV13 era, IPD cases among children with comorbidities (mainly immunosuppression due to a primary immunodeficiency or immunosuppressive or radiation therapies and chronic respiratory diseases, including asthma) caused by non-PCV13 serotypes accounted for approximately 60% of cases, of which 50% were PPV23-only and 50% were non-vaccine serotypes, predominantly serotypes with a lower invasive capacity (PPV23-only serotypes 11A and 15A and non-vaccine serotypes 6C, 23A, and 35B) [42,50]. Furthermore, vaccine failures were more common among individuals with at least one comorbidity and mainly associated with serotype 3, followed by serotypes 7F and 19A, highlighting the inadequate effectiveness of PCV13 among high-risk individuals [42,44]. In line with this, in the post-PCV13 era, among children and following transplantation in the United States, although the proportion of PCV13-serotype-caused IPD declined from 49% to 37% of IPD cases, 19A and 19F-associated cases were the most common breakthrough diseases, followed by PPV23-only serotypes (33F, 10, and 11) as well as NVTs (6C, 25B, and 35B) [41]. A meta-analysis in the United States and South Korea revealed that children with asthma vaccinated only with a PCV (PCV7, PCV10, or PCV13) but not with PPV23 had about 90% increased odds of IPD, mainly due to serotypes 4, 19F, and 9V (serotypes included in both PCV and PPV23), implying the added benefit of the combined PCV13/PPV23 vaccination schedule [40]. Unpublished data of Public Health England (United Kingdom) show that children with comorbidities, especially sickle cell disease, functional or anatomical asplenia, and diabetes, have higher mortality rates due to IPD than healthy children, while serotype-specific analysis showed that 19% of cases were caused by PPV23-only serotypes and 31% by non-vaccine serotypes [51]. A retrospective cohort analysis in the United Kingdom revealed that PPV23-only serotypes are more likely to cause IPD than the PCV13 serotypes and supported the evidence of higher rate ratios for IPD among asthmatic children and children with multiple at-risk conditions [46]. A small study in England among children with sickle cell disease, the majority of whom were appropriately immunized, revealed that the 15B/C serotype, a PPV23-only serotype, was responsible for most IPD cases [44]. In Brazil, a study among 51 pediatric oncology patients (only four vaccinated with at least a dose of PCV), PCV13 types 3 and 19A and PPV23-only serotypes 10A and 11A were the most frequent causes of IPD cases [47]. A more recent study in Denmark showed that following the introduction of PCVs in children, the net impact of serotype replacement varied considerably among different age groups and comorbidities. However, the relative increases in the incidence of IPD caused by specific NVT serotypes did not differ appreciably between risk groups in the post-vaccination era with the PPV23-only serotypes 8 and 12F, accounting for the majority of such cases [49].

The net benefits of conjugate vaccines remain significant among high-risk children in high-income countries with substantial reductions in IPD incidence rates; however, a rise in the proportion of PPV23-only and NV-serotypes causing IPD in high-risk individuals occurred following PCV13 implementation in these settings. Thus, the current disease epidemiology, characterized by a significant proportion attributed to PPV23-only serotypes, could justify the use of a combined PCV13/PPV23 immunization schedule for the protection of immunocompromised pediatric populations in the developed world. The rationale behind this schedule is to benefit from the improved opsonophagocytic antibody activity and the immunological memory provided by PCV13 as well as the complimentary broader serotype coverage provided by PPV23. However, vaccine immunogenicity and effectiveness in this population, and PPV23-driven immunological hyporesponsiveness, should be taken into account when drafting or recommending anamnestic PPV23 immunization policies for the “high-risk” [36].

### 1.2. Current Burden of Disease and Serotype Epidemiology in Low- and Middle-Income Countries

In low- and middle-income countries of Africa, South-East Asia, and Latin America, the incidence of IPD as well as the associated mortality are higher compared with high-income countries. In addition, the pneumococcal epidemiology in these settings is characterized by higher rates of carriage and transmission than in high-income settings [25,26,52,53,54], mainly due to the low vaccine coverage and higher prevalence of coexisting morbidities, such as human immunodeficiency virus (HIV) infection and sickle cell disease, that predispose to IPD [55]. In most low- and middle-income countries, PCV vaccines were introduced during the last decade, and the current main dosing schedule for PCV13 is (2 + 1) or (3 + 0), while that for PCV10 is (3 + 0) [56]. However, there are still several countries in Africa (e.g., Chad, Capo Verde, and South Sudan) and most countries in South-East Asia (e.g., Thailand and Indonesia) that have not included PCVs in their national vaccination program yet [57,58].

South Africa was one of the first African countries to introduce PCV7 immunization in 2009 and transitioned to PCV13 in mid-2011. The incidence of IPD decreased substantially following PCV implementation among vaccinated young children, but also in non-vaccinated adults through herd protection [59]. However, there is evidence of significant residual disease caused by serotypes 4, 19F, 19A, 3, and 1 in South Africa, while there was a rise in non-vaccine disease mainly in individuals older than 45 years of age led by the PPV23-only serotypes 8 and 12F, albeit not significant [60,61,62]. Even in South Africa, where PCVs were first implemented with the vaccine coverage reaching up to 99% in 2012 [62], there are not clear recommendations for vaccinating high-risk patients and there is no clear definition of patients at risk. Nevertheless, significant reductions in PCV13-type IPD have been documented after PCV implementation programs, although rates of IPD remained 25-fold higher in HIV-infected children than in HIV-uninfected children [59]. In the PCV13 era, among HIV-infected children, 11%, 17%, and 64% of IPD cases were caused by PCV7, PCV13-only, and non-vaccine serotypes, respectively. The most common non-vaccine types (NVTs) that emerged included 10A, 15A, 16B, and 35B, of which only 10A is included in PPV23, showing that the majority of non-vaccine serotypes currently causing IPD in the HIV-infected population are not included in PPV23 [63]. Moreover, in Latin America PCV13 serotypes 1, 3, 5, 6A/B, 7F, 9 V, 14, 18C, 19A/F, and 23F are most commonly associated with IPD among children and adolescents with predisposing risk factors, despite the high PCV13 coverage, implying the potential benefits of even higher rates of compliance with established vaccination schedule and its extension to high-risk individuals [64]. In Thailand, a country where PCVs are not included in the national vaccine program, the most common disease-causing serotypes are 6B and 23F, which are included in the PCVs, implying the potential benefit of its implementation in the future (VTs) [65].

Nasopharyngeal carriage of *S. pneumoniae*, which is an important precursor of pneumococcal disease, remains extremely common among children in many developing countries, such as India [66], Vietnam [67], Ethiopia, Mozambique, and Gambia [68], even with VTs (3, 11, and 19F), implying the need for higher vaccination coverage [69]. In South Africa, in spite of the high vaccine coverage, a high proportion of NVTs was reported; the most common NVTs were 15A, 15B/C 16F, 23A, and 35B, of which only 15B is included in PPV23, while a significantly lower proportion of VT carriage was observed in every age group of children under 5 years, highlighting the PCV effectiveness [69]. In Ghana, among HIV-infected children, the most prevalent carriage serotype was 19F, a PCV13 serotype, followed by 16F, a NVT serotype. Notably, the serotype coverage of PCV13 in this study was 41.5%; PPV23-only serotypes represented only 14.6%, and NVT serotypes 43.9% [70].

The pneumococcal disease serotype epidemiology among high-risk children is summarized in Table 3. Due to the limited data on pneumococcal disease, early currently available data on pneumococcal carriage that precedes pneumococcal disease are also described.

### 1.3. Data on PCV Effectiveness among High-Risk Pediatric Populations

PCVs have been universally used for pediatric populations since 2000 and their significant effectiveness among healthy children in high-income settings has been well documented. Early PCV7 studies had demonstrated 96% vaccine effectiveness against IPD among healthy children [75], while the effectiveness of different PCV13 schedules against IPD across high-income countries varies between 78% [76] and 89% [77].

Data on PCV effectiveness among high-risk individuals in high-income countries are limited. Thus, the recommendation of PCVs for the protection of individuals at increased risk for pneumococcal infection has been made based upon immunogenicity studies that showed non-inferiority to the previously recommended PPV23 [78]. An observational study conducted in the United States among children aged ≤ 10 years with SCD estimated the vaccine effectiveness against IPD to be 81% among those who received at least one dose of PCV7 [79]. In addition, it was shown that the effectiveness of one or more doses of PCV7 against vaccine serotypes was 96% in healthy children and 81% in those with coexisting disorders, underlying the significant benefit of conjugate vaccines in the protection of such populations [80].

Among healthy children in middle- and low-income countries, the introduction of PCV resulted in a significant reduction in deaths due to pneumococcal disease. Remarkably, Rwanda, Peru, and El Salvador, with the highest pneumococcal mortality rates in the pre-PCV era, had the greatest relative reductions ranging from 89% to 93% [81]. A 7-year population-based surveillance study in Gambia showed a 82% reduction in vaccine-type IPD and a 55% reduction in all-type IPD after the implementation of a 3 + 0 PCV13 schedule among children 0–2 years of age, while significant reductions were also observed in older children aged 2–4 years old [82].

Data on PCV effectiveness among high-risk children in developing counties are even more scarce. Nevertheless, there are some effectiveness and efficacy studies that collectively demonstrate that the PCV effectiveness for the prevention of IPD is higher than that of PPV23 in immunocompromised children, although inferior to that in the healthy aged-matched population [59,71,83,84]. An early study conducted by Klugman et al. in South Africa showed that a nine-valent PCV was 65% effective in preventing IPD in children with HIV infection [83]. This was further confirmed by post-licensure surveillance that showed an effectiveness of approximately 55% against IPD in HIV-infected children [84]. In South Africa, an 86% decline in PCV7-caused IPD was recorded among HIV-infected children < 2 years of age [71]. Moreover, a recent study by Cohen et al. demonstrated that two or more doses of PCV13 have an effectiveness of 91% against vaccine-type IPD among children with HIV [59]. PCV13 implementation in the South African EPI (2 + 1 schedule) was significantly effective against VT-caused pneumococcal disease in children not infected with HIV (against 19A, 14, and 23F), in children exposed to HIV but not infected, as well as in malnourished children, given that the latter two are considered important risk factors for IPD [59]. In Malawi, a lower possibility of VT carriage was reported among vaccinated children aged 3–5 years as well as a more marked decline in VT carriage among unvaccinated children 6–8 years old due to a herd protection effect [71].

Thus, early surveillance data from low-income countries that have already incorporated PCV vaccination show indeed a high degree of vaccine effectiveness with a significant public health benefit through direct and herd protection [36]. The benefit of such significant reductions in pneumococcal disease is expected to extend to the large numbers of children with comorbidities living in these regions. Notably, co-existing morbidities such as malaria, HIV, and sickle cell disease in these settings may also reversely affect vaccine efficacy. As for children with functional asplenia or splenectomy due to sickle cell disease, vaccine efficacy and immunogenicity studies have shown significant results, albeit to a lesser extent compared with the general population [85].

### 1.4. Data on PPV23 Effectiveness among High-Risk Pediatric Populations

PPV23’s effectiveness against IPD among high-risk individuals remains debatable despite its extensive use for over 25 years. A number of randomized controlled trials and observational studies [86,87,88], as well as several meta-analyses [89,90,91], have been conducted among children and adults with immunocompromising conditions with inconclusive results regarding vaccine effectiveness against IPD and pneumonia. Nevertheless, the most recent Cochrane meta-analysis demonstrated effectiveness of PPV23 against IPD for healthy adults but no protection against pneumonia and all-cause mortality [92]. Moreover, a sub-analysis in high-risk populations of high-income countries showed no evidence of protective vaccination efficacy even against IPD. Interestingly, there are findings implying not only attenuated protection but possibly an increased risk for IPD in individuals with severe immunodeficiency induced by the use of polysaccharide vaccines [36]. Similarly, increased rates of all-cause pneumonia have been demonstrated among HIV-infected PPSV23 recipients in Uganda compared with unvaccinated patients [93]. Moreover, evaluation of the kinetics of PPV23-induced serotype-specific antibodies in high-risk individuals revealed that antibody levels decrease substantially shortly after vaccination, implying that the duration of protection is relatively short-lived [60,94,95]. According to a study among children with chronic disease, the effectiveness against invasive disease caused by PPV23 serotypes was 63%, and the effectiveness against PPV23-only serotypes was 94% [89]. Another study in children aged > 5 years at risk for serious pneumococcal infection showed that the overall PPV23 efficacy for preventing infection caused by serotypes included in the vaccine was 57% [37]. In an effort to protect individuals at risk, immunization with PPV23 used to be repeated every 5 years. However, the repeated use of PPV23 required for protection of high-risk individuals has been associated with hyporesponsiveness [36].

## 2. Discussion

This is a time of change for pneumococcal vaccination policies in both the developed and the developing world. In Europe, there is a continuing effort to implement infant schedules with a reduced number of doses in order to minimize public expenses and facilitate the funding of other important vaccines [96]. On the other hand, countries in Africa, South-East Asia, and other low-income regions are currently introducing a universal PCV infant schedule, expecting significant changes in disease burden and serotype distribution. In this study, we aimed to summarize the current knowledge regarding the pneumococcal disease burden and vaccine effectiveness against IPD among high-risk individuals in high- and low-income countries in order to assess the optimal immunization policies for the protection of high-risk individuals in each setting.

In the developed world, disease epidemiology and vaccine efficacy data support the recommendation of a combined PCV13/PPV23 immunization schedule for immunocompromised individuals [22]. PCV13/PPV23 immunization schedules have been established on the basis of combining PCV13-induced immunological memory with the broader serotype coverage of PPV23. Today, sustaining high coverage rates of the direct PCV13 immunization of high-risk individuals is crucial, as reduced-dose infant schedules might lead to attenuation of herd protection [97]. Regarding PPV23, current surveillance data in the developed world show that PPV23-only serotypes take up a significant percentage of IPD cases among the high-risk population; thus, the continuation of one dose of PPV23 after PCV13 is expected to offer some additional benefit, despite the documented limited effectiveness of the vaccine in such individuals. However, PPV23 should be used with caution, as it could attenuate PCV13-induced immunological memory if administered shortly before or after the conjugated vaccine [36].

In the low-income regions, the success of the early years of PCV implementation should be enhanced by maximizing coverage rates among infants. Regarding the use of PPV23 for the protection of high-risk individuals in low-income countries, there is currently no clear evidence of emerging PPV23-only serotype IPD in these settings, implying that the potential benefits of PPV23 implementation may not offset its limitations (low effectiveness among high-risk individuals, the hyporesponsiveness phenomenon). As for HIV-infected individuals in developing countries, the WHO has concluded that currently available evidence does not support the routine use of PPV23 in HIV-infected patients and focus should be placed on the early HAART initiation [98] in order to maintain the ability to mount protective immune responses.

As in most low-income countries, high-risk older children and adults are not universally immunized with PCVs, so their protection largely depends upon the establishment of strong herd protection through the infant vaccination schedules. Therefore, the optimization of infant schedules in order to maximize the conferred herd protection is highly important. Recent data show that the lack of anamnestic immunization in the three-dose schedule has a significant effect on both direct and indirect vaccine protection. More specifically, the 3 + 0 PCV13 schedule implemented in Australia [99] resulted in lower disease reductions than the 2 + 1 schedule in the United Kingdom [100] among unvaccinated adults aged 15–44 years and the >65 year olds. This observation is further supported by epidemiological data regarding pneumococcal serotype 1 meningitis outbreaks in Ghana, where 3 + 0 schedules have been adopted. Currently, pneumococcal meningitis outbreaks continue to occur in this region, despite the introduction of a 3 + 0 PCV13 infant schedule in 2012 and the achievement of high vaccination coverage. Although PCV13 vaccination seems to have protected infants and young children under 5 years of age in the most recent outbreak of 2016, older children and adults were significantly affected, suggesting that the implemented 3 + 0 schedule did not offer protective herd protection to these older age groups and further justifies the need for direct protection through immunization of these high-risk groups [60]. Moreover, vaccination policy-makers should revisit the possibility of introducing 2 + 1 immunization schedules across the African Meningitis belt in order to maximize cost-effectiveness across all age groups and stop vaccine-type outbreaks. Given the likely importance of an early reduction in transmission intensity to maintain a reduced carriage prevalence, a catch-up-campaign with booster doses over a broader age range (e.g., <5 years of age) may also be required. Although GAVI has considerably reduced PCV costs for low-income countries [101,102], the vaccine impact must be optimized (particularly indirect effects) to achieve financial sustainability.

Therefore, a combined PCV/PPV23 schedule may be justified for use among high-risk individuals in developed countries. In these combined schedules, where recommended, PCV administration should precede PPV23 administration, in order for the PCV to establish immunological memory for the serotypes it includes and for PPV23 to induce antibody responses to the additional serotypes [36]. However, there are increasing data showing that PPV23 may reversely affect the immunological memory induced by the PCV even when it is given soon after PCV13 [36]. Importantly, it has been well-documented that hyporesponsiveness is a time-dependent phenomenon and PCV-induced memory is affected more significantly when PCV13 is given shortly after PPV23 [36]. However, current guidelines still allow for short intervals between PPV23 vaccination and subsequent PCV13 vaccination for PPV23-experienced individuals; for children previously immunized with PPV23, a single PCV13 dose given just ≥8 weeks after the last PPV23 dose is recommended [103]. Measurement of levels of serotype-specific antibodies before vaccination could play a beneficial role in optimizing intervals between vaccinations for such individuals. In the presence of high antibody levels, subsequent pneumococcal vaccination may be delayed in order to maximize the potential of the immune response to future vaccination in terms of magnitude and longevity.

On the other hand, in the developing world emphasis should be placed on better compliance with PCV vaccination schedules. Nevertheless, all currently available immunization strategies for high-risk individuals have limitations due to reduced vaccine efficacy and the unique epidemiology of pneumococcal disease among patients at risk.

To expand serotype coverage, a 20-valent PCV (PCV20) containing PCV13 components and seven additional serotypes (8, 10A, 11A, 12F, 15B, 22F, and 33F) has been recently developed and already approved for use among adults > 18 years of age. These serotypes were selected due to their association with increased disease severity, invasive potential, antibiotic resistance, and increased prevalence as a cause of pediatric pneumococcal disease worldwide in the post-PCV era [20]. A meta-analysis has highlighted that these seven serotypes are among the most prevalent serotypes causing pediatric IPD in countries with ongoing PCV programs [20,104]. Surveillance data by the Centers for Disease Control and Prevention found that in 2018, these seven serotypes alone accounted for an estimated 37% of IPD in U.S. children < 5 years of age [105] PCV20 implementation for high-risk children is expected to further enhance their protection against pneumococcal disease, especially in the developed world where the serotype replacement phenomenon is more obvious. However, pneumococcal conjugate vaccine formulations designed exclusively for high-risk patients might be inevitably necessary while waiting for the development of innovative protein-based vaccines that could offer serotype-independent coverage and an optimal immunological profile for individuals at risk. Still, since the type and invasive potential of new serotypes are not easily predictable, careful monitoring of pneumococcal disease is necessary.

## 3. Conclusions

In the developed world, a combined PCV/PPV23 schedule may be justified for use among high-risk pediatric populations based on serotype-epidemiology. However, reassessment of the intervals between vaccinations is an important priority to maximize the potential of the immune response to future vaccination in terms of magnitude and longevity.

In the developing world, special focus should be given on better compliance with PCV vaccination schedules. In these settings with higher prevalence of coexisting morbidities, such as human immunodeficiency virus (HIV) infection and sickle cell disease that predispose to IPD, current serotype epidemiology does not justify PPV23 administration, as its limitations will most likely outweigh its benefits.

## Figures and Tables

**Table 1:** Pneumococcal vaccines and the serotypes covered by each of them.

Vaccine	Serotypes Covered
PCV7	4, 6B, 9V, 14, 18C, 19F and 23F
PCV10	1, 4, 5, 6B, 7F, 9V, 14, 18C, 19F and 23F
PCV13	1, 3, 4, 5, 6A, 6B, 7F, 9V, 14, 18C, 19A, 19F and 23F
PPV23	1, 2, 3, 4, 5, 6B, 7F, 8, 9N, 9V, 10A, 11A, 12F, 14, 15B, 17F, 18C, 19A, 19F, 20, 22F, 23F and 33F

**Table 2:** Summary of studies showing the pneumococcal disease serotype epidemiology among pediatric populations at risk in the developed world.

Study	Country	Vaccination Schedule	High-Risk Factor	Pneumococcal Disease/ Carriage	Age	Serotypes Recorded
Ladhani et al. (2013) [5]	UK	PCV	Children with comorbidities	IPD	3–59 months	1, 3, 5, 6A, 7F, 19A/PPV-23 only (2, 8, 9N, 10A, 11A, 12F, 15B, 17F, 20, 22F, 33F)/remaining non PPV-23 serotypes (all other serotypes)
Castro-Rodriguez et al. [40]	USA	PCV13	Children with asthma	IPD	0–18 yers	19F, 4, 9V
Olarte et al. (2016) [41]	USA	PCV13	Children following transplant	IPD	≤18 years	19A, 19F, 33F, 10, 11, 6C, 26B, 35B
Lapidot et al. (2020) [42]	USA	PCV13	Children with underlying comorbidities (cerebral palsy, chronic lung disease, congenital heart disease, prematurity/low birth weight, and sickle cell disease)	IPD	<18 years	3
Yildirim et al. (2020) [43]	USA	PCV13	Any underlying risk factor- 69.2% of mortality cases had a comorbidity (sickle cell disease, hematological malignancy, neuromuscular disorder, chronic lung disease, congenital heart disease)	IPD	<18 years	PCV13 serotypes (1, 3, 4, 5, 6A, 6B, 7F, 9V, 14, 18C, 19A, 19F, 23F), NVTs (all other serotypes)
Yildirim et al. (2015) [44]	USA	PCV13	Children with underlying medical conditions	IPD	<18 years	PPV-23 only serotypes (2, 8, 9N, 10A, 11A, 12F, 15B, 17F, 20, 22F, 33F)/serotypes not icluded in any of the vaccines (6C, 23A, 11A, 35B, 15A, 15C)
Oligbu et al. (2017) [45]	UK	PCV13	Twelve children with sickle cell disease (eleven homozygote for hemoglobin S (HbSS) and one double heterozygote for hemoglobin S and C (HbSC))	IPD	<5 years	7F, 15A, 15B/C, 35B, 35F
Pelton et al. (2014) [46]	UK	PCV ± PPV23	Children with chronic medical conditions	IPD	<18 years	PCV7 (4, 6B, 9V, 14, 18C, 19F, and 23F) and PCV13 serotypes (1, 3, 4, 5, 6A, 6B, 7F, 9V, 14, 18C, 19A, 19F, and 23F)/PPV23-only (2, 8, 9N, 10A, 11A, 12F, 15B, 17F, 20, 22F, 33F)
Lages et al. (2020) [47]	Brazil	PCV	Pediatric oncology patients (POP)-(n = 51)	IPD	<18 years	3, 19A, 10A, 11A
Asner et al. (2019) [48]	Switzerland	PCV	Healthy children and children with a risk factor for IPD	IPD	<17 years	PCV13 serotypes 3, 7F, 19A)/non-PCV serotypes (15, 23)
Weinberger et al. (2019) [49]	Denmark	PCV	Children with and without comorbidities	IPD	<5 years	PCV7 (4, 6B, 9V, 14, 18C, 19F, and 23F) and PCV13 serotypes (1, 3, 4, 5, 6A, 6B, 7F, 9V, 14, 18C, 19A, 19F, and 23F)/non-PCV 7/13 serotypes (6A, 6C)

**Table 3:** Summary of studies showing the pneumococcal disease serotype epidemiology among pediatric populations at risk in the developing world.

Study	Country	Vaccination Schedule	High-Risk Factor	Pneumococcal Disease/Carriage	Age	Serotypes Recorded
Cohen et al. (2017) [59]	South Africa	PCV13	HIV	Pneumococcal infection IPD	<5 years	19A
Falleiros-Arlant et al. (2015) [64]	Latin America	PCV PCV13	children and adolescents with predosposing risk factors	IPD Pneumonia	5–19 years	PCV13 serotypes (1, 3, 4, 5, 6A, 6B, 7F, 9V, 14, 18C, 19A, 19F, and 23F)
Sutcliffe et al. (2019) [66]	India	PCV	-	Clinical pneumonia	2–59 montths	6A, 6B, 14, 19A, 19F
Nguyen et al. (2019) [67]	Vietnam	PCV	Acutespiratory infection (ARIs)	Pneumococcal disease	<5 years	19F
Usuf et al. (2007) [68]	Ethiopia, Mozambique, Gambia	PCV	-	Pneumococcal disease IPD	<5 and 5–15 years	19F, 6B, 6A, 14, 23F
Donkor et al. (2017) [70]	Ghana	PCV PCV13	HIV	Pneumococcal disease	<15 years	19F, 6F
Swarthout et al. (2020) [71]	Malawi	PCV	HIV	IPD	<10 years	PCV13 serotypes (1, 3, 4, 5, 6A, 6B, 7F, 9V, 14, 18C, 19A, 19F, and 23F), non-PCV13 serotypes (all other serotypes)
Kartasasmita et al. (2020) [72]	Indonesia	PCV	HIV	Pneumococcal disease	4–144 months	PCV13 serotypes (3, 6A, 6B, 14, 19A,19F, 23F), non-PCV13 serotypes (11A, 15B/C, 23A)
Mackenzie et al. (2012) [73]	Gambia	PCV	-	IPD	2–59 months, >5 years	1, 3, 5, 6A, 7F, 19A
Ramakrishnan et al. (2010) [74]	Nigeria, Senegal, Kenya, Congo	PCV	Sickle cell disease	Pneumococcal infection	0–168 months	PCV7 serotypes (4, 6B, 9V, 14, 18C, 19F, and 23F), non-PCV7 serotypes (all other serotypes)

## Data Availability

Not applicable.
